# Dendritic Cell-Based Graft Tolerance

**DOI:** 10.5402/2011/347134

**Published:** 2011-04-10

**Authors:** Mnasria Kaouther, Oueslati Ridha

**Affiliations:** Laboratory of Environnemental Immuno-Microbiology and Cancerogenesis (IMEC Unit), Department of Biology, Faculty of Sciences of Bizerte, Zarzouna 7021, Tunisia

## Abstract

It has recently been demonstrated that mouse and human dendritic cells (DCs) can produce IL-2 after activation. However the role of the IL2/IL2R pathway in DC functions has not yet been fully elucidated. The results presented in this study provide several new insights into the role of this pathway in DCs. We report that stimulation of human monocyte-derived DCs with LPS strongly upregulated CD25 (**α** chain of the IL2R) expression. In additon, by using a humanized monoclonal antibody against CD25, we demonstrated that the IL2 signalling in DC upregulated both IL-12 and **γ**IFN production but decreased IL10 synthesis. We also found that LPS-matured DCs produced IL2. Taken together, these results suggest that IL-2 actively contributes to the DC activation through an autocrine pathway. Furthermore, our results indicate that the IL2 pathway in DC is involved in the development of T-helper priming ability and in the upregulation of surface markers characteristic of a “mature” phenotype. This study therefore provide new molecular clues regarding the split between these two phenomena and unravel new mechanisms of action of anti-CD25 monoclonal antibodies that may contribute to their action in several human immunological disorders such as autoimmune diseases and acute allograft rejection.

Dendritic cells (DCs) are bone-marrow-derived cells that populate all lymphoid organs as well as nearly all nonlymphoid organs. Although DCs display a heterogeneous group of cells that represent differences in origin, anatomic, location, cell surface phenotype, and function. They are powerful antigen-presenting cells that have a critical role in the initial activation of naïve cells and the recall of memory immune responses. 

Immature DCs reside as sentinels in non lymphoid organs; they are high adapted for the uptake of antigen via receptor- and nonreceptor-mediated mechanisms and readily degrade antigens in endocytic vesicles to produce antigen peptides capable of binding to Human Leukocyte Antigens class II. 

Upon maturation with pathogens, activated T Lymphocytes and/or inflammatory signals such as TNF*α*, IL-1*β*, or lipopolysaccharides (LPS), immature DCs underwent genetic reprogramming leading to mature DCs characterized by high expression of HLA class II molecules absence of lineage markers such as CD14 (monocytes), CD3 (T cells), CD19, CD20 (B cells), CD56 (NK cells), high level expression of costimulatory molecules CD83, CD86, CD80, CD40, and adhesion molecules such as CD11a, CD11c. Mature DCs also acquire ability to migrate which is regulated by expression of chemokines and chemokines receptors CCR7.

These chemokines guide mature DCs to lymphatic vessels and to secondary lymphoid organs.

**DC Subsets**

In human DCs comprise at least three distinct subsets.

- Langerhans cells LC and interstitial DCs belonging to the myeloid lineage and plasmacytoid DCs originate from a lymphoid precursor (Table 1).
- LCs are localized in the layers of the epidermis in the skin and other mucosal areas, whereas interstitial DCs are present in the dermis of the skin and in most other organs.
- Human plasmacytoid DCs are found in the T-cell zones of lymphoid organs (Table 1).

There seem to be several pathways to generate DCs.

 Blood monocytes give rise to DCs when cultured with the appropriate cytokines. DCs progenitors are also present in bone marrow. CD34^+^ subsets of haematopoietic progenitors give rise to all blood cells and DCs.

In vitro human DCs can be generated from CD34^+^ bone marrow and peripheral blood progenitor's cells: after culture with different cytokine combinations including GM-CSF, TNF*α*, CD40L. Alternatively, DC can be derived from CD14^+^ peripheral blood adhering monocytes. 

PBMCs are obtained by cytapheresis isolated by Ficoll Hypaque density gradient and cultured with GM-CSF and rhIL-4. At the end of 5-days culture, immature DCs are harvested. Maturation was induced by adding proinflammatory stimuli such as TNF*α*, LPS, or IL-1*β* or on ligation of CD40. At the last 48 h of culture on day 7, mature DCs were harvested (Figure 1).

Upon maturation, DCs display high expression of costimulatory, presenting and accessory molecules, uptake molecules were decreased. Induction of allogeneic T-cell proliferation in MLR by DCs has been associated with DC maturation including production of proinflammatory cytokines such as TNF*α*, IL-12, IFN*δ*. Alternatively, DCs produce low levels of IL-10, anti-inflammatory cytokine. 

Recently, it has been reported that upon activation DCs exhibit transient production of IL-2 and express IL-2R*α*, a property that appears to be related to their capacity to initiate immune responses. Besides, the ability of DCs to produce IL-2 after encountering inflammatory stimuli provides the first crucial signals for the activation of naïve T cells. The kinetics of IL-2 production by DCs are compatible with the appearance of HLA class II and class I peptides at the cell surface of DCs, so IL-2 appears to be one of the key molecules conferring unique T-cell stimulating capacity on DCs. 

Dendritic cells (DCs) play a key role in the initiation and regulation of both the adaptative and the innate immune responses [1–5]. DC progenitors leave the bone marrow (BM) and give rise to circulating precursors. They differentiate into immature DCs that are distributed throughout the peripheral tissues and mucosa and act as sentinels of the immune system [3, 4]. They are characterized by a high capacity for antigen uptake and processing [3]. In response to inflammatory stimuli, immature DCs rapidly undergo a complete genetic reprogramming. During the first twenty-four hours, DCs experience all the transcription modifications necessary to progress from immature to mature cells that are characterized by a high capacity for antigen presentation and T-cell priming [5, 6]. The mature DCs then migrate to the secondary lymphoid organs where they interact with T cells. This complex “maturation” process involves not only the upregulation of costimulatory surface proteins and the optimization of antigen presentation capacities, but also the production of cytokines and chemokines [4–6]  that profoundly influences the outcome of the T-cell response. The signals in DC involved in each of these phenomena are not completely understood.

The signals delivered by DC are believed to direct the T-cell response into either a Th1, Th2, or nonpolarized T-cell response [7, 8], but they can also drive the differentiation of regulatory T cells involved in self-tolerance [2–9]. Thus, the extraordinary versatility of dendritic cells has recently become apparent. Although much is known about the cytokines produced by T cells and the T-cell-associated transcription factors that determine the T-helper polarization, the early “decision-making mechanisms” which result in a given type of immune response are poorly understood and possibly are based on specific cytokine and chemokine synthesis profiles by DC. This area remains a subject of intense investigations.

In addition to affecting adaptative immunity, the cytokine synthesis profiles of DCs might also influence their capacity to activate cells involved in innate immunity such as NK cells [10, 11]. 

It has recently been demonstrated that mouse and human DCs can both produce IL-2 after activation with several stimuli [12]. The expression and the function of the IL2 receptor on DCs have been rarely studied. One study in mice showed IL-2R*α* expression on DCs but they could not ascribe any role to its expression [13]. Thus, the role of IL2 signalling in DC function remains poorly defined. It was, therefore, relevant to study how this pathway influenced the DC cytokine secretion profile and their ability to activate CD4+T cells.

In the present study, we report that CD25 expression was strongly upregulated on DCs by several maturation signals such as LPS. In addition, we demonstrated that IL2 signalling in DCs upregulated both IL-12 and IFN*γ* production but decreased IL10 synthesis. We also found that LPS-matured DCs produced IL2. Taken together, these results suggest that IL-2 actively contributes to DC activation through an autocrine pathway. Finally, our results indicate that the IL2 pathway in DC is involved in the development of T-helper priming ability and in the upregulation of surface markers characteristic of a “mature” phenotype. Antagonizing IL2 signalling in DCs may therefore influence the immune response in vivo. These actions of anti-CD25 antibodies on DC functions may contribute to their action *in vivo* on several immunological disorders such as autoimmune diseases [14, 15] and prevention of acute human allograft rejection [16].

In conclusion, our data suggested that the level of IL2 signalling in DCs regulate their capacity to efficiently prime the T-helper immune response. Therefore, targeting this pathway ex vivo might be useful for protocols in cellular therapy to orientate DC toward a tolerogenic profile (by using antiCD25 treated DC) able to promote Treg differentiation or at the opposite to promote or restore immunity against tumors (by using IL2-treated DCs).

## Figures and Tables

**Figure 1:**
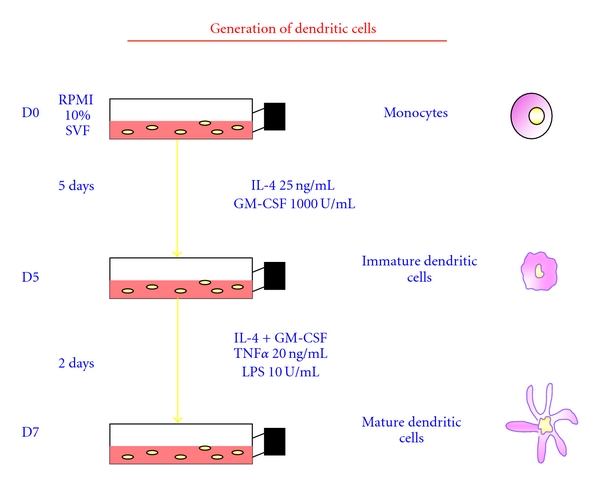
Protocol of dendritic cell generation.

**Table 1:** 

Postulated lineage	Lymphoid	Myeloid	
DC subtype	Plasmacytoid DC	Interstitial DC	Langerhans cell
*Fully differentiated DC*	CD11c^−^ CD123^+^	CD11c^+^ CD123^−^	CD11c^+^ CD123^−^
	CD11b^−^ CD13^−^ CD33^−^	CD11b^+^ CD13^+^ CD33^+^	CD11b^+^ CD13^+^ CD33^+^
	CD1a^−^	CD1a^−^	CD1a^+^

*Localization*	T-cell zones of lymphoid organs	T-cell zones of lymphoid organs immature cells in peripheral tissues	T-cell zones of lymphoid organs immature cells in epithelia

*Function*			
(i) Mannose receptor-mediated endocytosis	—	+ + +	—
(ii) IL-12	+ + +	+ + +	+ + +
(iii) Il-10	—	+ + +	—
(iv) IFN*γ*	+ + +	—	—
(v) CD4+ T-cell priming	+ + +	+ + +	+ + +
